# Correlation of CT texture changes with treatment response during radiation therapy for esophageal cancer: An exploratory study

**DOI:** 10.1371/journal.pone.0223140

**Published:** 2019-09-26

**Authors:** Zhumin Yan, Jingqiao Zhang, Hai Long, Xueming Sun, Dingjie Li, Tian Tang, X. Allen Li, Wu Hui

**Affiliations:** 1 Department of Radiation Oncology, Affiliated Cancer Hospital of Zhengzhou University, Zhengzhou, China; 2 Department of Radiation Oncology, New York Proton Center, New York, New York, United States of America; 3 Department of Radiation Oncology, Medical College of Wisconsin, Milwaukee, Wisconsin, United States of America; Chang Gung Memorial Hospital at Linkou, TAIWAN

## Abstract

**Purpose:**

To analyze the change of CT texture features of esophageal squamous cell carcinoma (ESC) during RT delivery and to correlate these changes with the RT responses and survival.

**Methods:**

A total of 61 ESC patients received radical RT were screened. Weekly CTs (4–6 sets for each patient) were acquired during RT. The tumors, normal esophageal mucosa tissue (NEC) of 5 cm and the spinal cord in the relevant area were delineated. CT texture features were extracted with a home-made tool. The changes of these features were analyzed by t-test. The correlations of the changes of features with RT responses and with patient survival were investigated by Pearson analysis.

**Results:**

The average changes were increased by 0.00072 ±0.00197 for coarseness, by 0.14 ±0.40 for entropy, and by 2.34 ±3.56 for strength. In addition, the average changes were reduced by 8.88 ±15.71cc for volume and by 0.07 ±0.11 for busyness. The changes of the coarseness, strength, STD and entropy in ESC were different for the good and poor response groups. The survival rate of the patients was significantly correlated with the change of coarseness and strength (*P* = 0.0027 and *P* = 0.0001).

**Conclusions:**

During RT, changes of CT texture features of ESC, e.g., coarseness, strength, STD, entropy and volume are correlated with radiation response and survival rate. With more clinical data and robust research, CT features, e.g., coarseness and strength, can be selected as outstanding imaging biomarkers for prediction of RT prognosis of ESC.

## Introduction

Esophageal carcinoma is a common malignant tumor in China. Although the incidence and mortality of esophageal carcinoma in China have declined in recent years, esophageal carcinoma still is a major world health problem. Radiation therapy is one of the main treatments for esophageal carcinoma. Despite technological advances in radiotherapy for esophageal cancer, such as three-dimensional conformal radiotherapy and intensity-modulated radiotherapy, the 5-year survival rate is less than 30%[1–3].

Radiomics, describes tumor phenotype objectively and quantitatively through high-throughput extraction of quantitative features[4,5]. Medical imaging, such as CT, PET-CT and MR images was used to definite tumor diagnosis, evaluate treatment response and tumor target delineation by collecting imaging data[6–9]. During radiation therapy, CT provides high quality imaging that guides radiation delivery and monitors tumor response, which was widely used in clinic. It has been reported that the CT number (CTN), measured in Hounsfield units (HU), can be used to evaluate the treatment response of lung and liver tumors as well as normal tissue[10–13]. Daily diagnostic-quality CT images obtained during radiotherapy treatments have been used to observe changes in tumors and selected OARs of head&neck and pancreatic cancer[14,15]. It has been reported that CT texture analysis was used to find one or a set of radiomics indicators that can predict tumor treatment response and survival of patients with esophageal cancer[16,17]. However, there is no common standard at present.

In this study, the daily diagnostic quality CT data of ESC acquired during routine CT-guided RT delivery was quantitatively analyzed. The radiomics metrics, including mean CT number (MCTN), volume, standard deviation (STD), skewness, kurtosis, entropy, coarseness, busyness, complexity and strength were investigated to evaluate treatment prognosis.

## Materials and methods

### Patient data

Diagnostic-quality CT images for 61 patients between January 2013 and December 2017 with stage II-IV (staging system, American Joint Committee on Carcinoma, 7th edition) squamous cell carcinoma of esophagus were acquired from a CT-on-rails (CTVision; Siemens Medical Solutions USA, Malvern, PA) during daily CT-guided IGRT. Among the 61 patients, 39 were male and 22 were female. The patient ages ranged from 46 to 85, with a median of 70. Radiotherapy doses ranged from 32 to 66 Gy, of which 2 (patient 3 and patient 42) were preoperative radiotherapy (radiation dose 36Gy in 20 fractions). 42 patients accepted 2 to 3 cycles of cisplatinbased chemotherapy concurrently. General information about the patients are summarized in Table 1. This study defined (CR) as good response (22 patients) and (SD) as poor response (10 patients).

**Table 1:** Patient characteristics, treatment methods and outcome data.

Patient no.	Sex	Tumor Stage	Radiation dose (Gy)	Fractions	Chemotherapy?	Efficacy	Survival time(m)
1	M	III	2.00	30	Yes	CR	65
2	F	III	1.80	30	Yes	SD	6
3	M	II	1.80	20	Yes	PR	67
4	M	III	1.80	35	No	PR	30
5	M	II	1.80	35	Yes	PR	39
6	M	III	1.80	33	Yes	PR	10
7	F	IV	1.80	35	No	PR	56
8	F	II	2.00	32	Yes	PR	12
9	F	III	1.85	33	Yes	PR	12
10	M	II	2.00	33	Yes	SD	33
11	M	III	2.00	30	No	CR	13
12	M	II	2.00	25	Yes	CR	42
13	M	III	2.00	33	Yes	PR	35
14	M	II	1.80	33	No	PR	38
15	F	III	2.00	33	Yes	CR	31
16	M	II	2.00	30	Yes	CR	51
17	M	II	2.00	32	No	CR	13
18	F	III	2.00	28	Yes	PR	33
19	M	II	2.00	30	No	SD	3
20	M	II	1.80	33	Yes	PR	2
21	M	II	2.00	33	Yes	CR	69
22	M	II	2.00	30	No	CR	67
23	F	III	2.00	30	Yes	PR	18
24	M	II	2.00	30	Yes	CR	50
25	M	II	2.00	30	No	PR	19
26	F	IV	1.86	35	No	CR	53
27	M	II	1.86	35	No	PR	46
28	F	IA	2.00	30	Yes	CR	56
29	F	IV	2.00	31	Yes	PR	60
30	F	II	2.00	33	Yes	CR	70
31	F	III	1.85	33	Yes	PR	55
32	M	IV	2.00	25	No	SD	11
33	M	III	1.80	33	No	PR	33
34	F	III	1.85	33	No	CR	34
35	F	II	2.00	32	Yes	CR	69
36	M	III	2.00	30	Yes	CR	67
37	M	III	2.00	25	No	CR	30
38	M	IV	2.00	33	Yes	SD	35
39	M	IV	1.80	33	Yes	PR	13
40	M	IV	2.00	30	No	PR	12
41	F	II	1.80	33	Yes	PR	7
42	F	III	1.80	20	Yes	PR	21
43	M	II	2.00	30	Yes	PR	5
44	F	III	2.00	30	Yes	PR	7
45	M	III	2.00	33	Yes	PR	63
46	M	III	2.00	30	Yes	PR	32
47	M	II	2.00	33	No	SD	12
48	F	IV	2.00	30	Yes	PR	63
49	F	II	2.00	30	Yes	CR	22
50	M	IV	2.00	30	Yes	PR	13
51	M	IV	1.80	23	Yes	SD	29
52	M	III	2.00	16	Yes	SD	3
53	M	IV	1.80	33	Yes	PR	18
54	F	II	1.80	33	Yes	CR	59
55	M	II	2.00	30	Yes	CR	58
56	M	III	1.80	36	Yes	CR	48
57	M	II	2.00	30	Yes	CR	80
58	M	III	2.00	30	No	SD	11
59	M	II	1.80	33	Yes	SD	8
60	F	III	1.80	33	No	PD	33
61	F	III	1.80	33	No	CR	48

M: male; F: female; CR: complete remission; PR: partial remission; SD: stable disease; PD: progressive disease.

This study has been examined and approved by the Ethics Committee of Cancer Hospital affiliated to Zhengzhou University. Because this study is a retrospective study, we provide a template for informed consent to treatment. However, it was signed by the patient himself or the patient's family before receiving treatment.

### Daily CT data

All daily CT images were acquired using a CT-on-rails with the normal acquisition mode(120kVp, image noise ±1 Hounsfield unit [HU] and slice thickness 4mm). The patients were immobilized with a thermoplastic mask. For each case studied, daily CT images for analysis included the first to the last fractions, and one fraction per week, with total of 4–6 CT sets. In this study, ESC referred to a lesion area containing no air cavity esophageal wall with thickness ≥ 10 mm. The ESC delineation of the first fraction was based on the patient's MRI and esophageal endoscopic findings. ESC, NEC and spinal cord were contoured by using a commercial tool (MIM Software Inc 5.4.2.C328-07) on daily CT images. All contours were drawn by the same doctor and reviewed by experienced radiation oncologists.

### Data analysis

An in-house software tool, CUBETAB developed with MATLAB (version R2017b; Math Works, Natick, MA) was used to analyze the daily CTs with the generated daily contours of ESC, NEC and cord. The differences between CT texture features or two patient groups were tested using the Student’s paired t-test with a two tailed hypothesis. Pearson analysis was used to analyze the association between texture features and survival. For all statistical analyses, *P* < 0.05 was considered significant.

Evaluation criteria for measurable lesions in WHO solid tumor efficacy evaluation criteria (WHO two-path measurement method)[18] was used to evaluate the early RT response. CT, MRI, and esophageal angiography were used to monitor the response of patients with esophageal carcinoma after chemoradiotherapy.

## Results

### Changes in CT number of images

**Fig 1:**
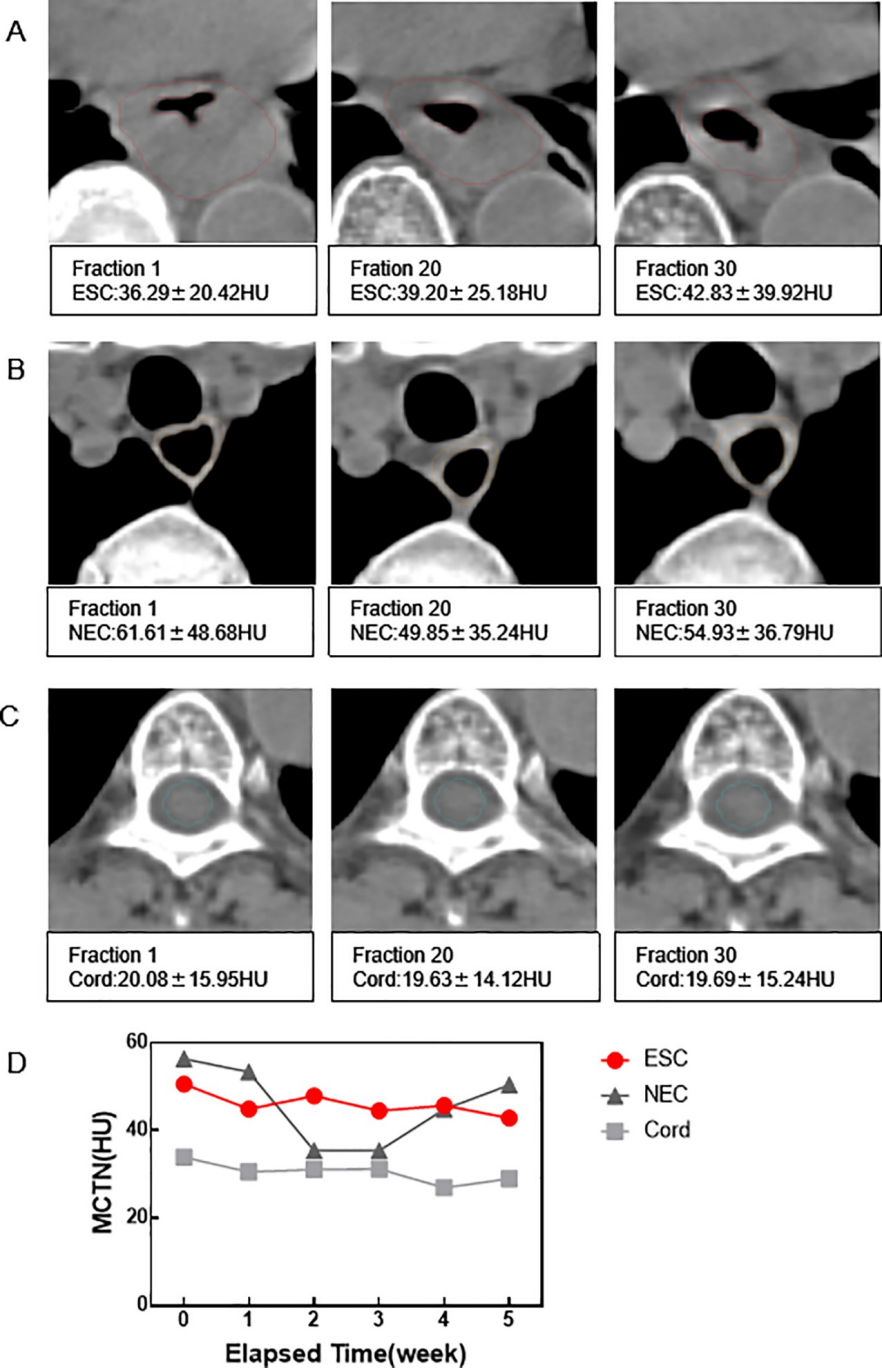
CTN changes of patient 27. Comparisons of ESC (A), NEC (B) and cord (C) for the 1^st^ (no radiation received),20^th^,and 30^th^ fraction. MCTN changes in ESC, NEC and cord with the elapsed time were shown in (D).

As shown in Fig 1(A), the ESC volume shrank significantly, while the NEC and cord changed little in Fig 1(B) and 1(C). For the case (Patient 27), the gross change of MCTN (the MCTN of last week minus the first week) in ESC decreased by 7.74 HU (Fig 1(D)), which showed an oscillating pattern during the treatment. While the weekly change of MCTN in NEC decreased significantly in the 2^nd^ to 3^rd^ weeks during radiotherapy, then increased to the value of little change from the 1^st^ treatment. There was no significant change in the spinal cord.

### Correlation between texture features and treatment response

**Fig 2:**
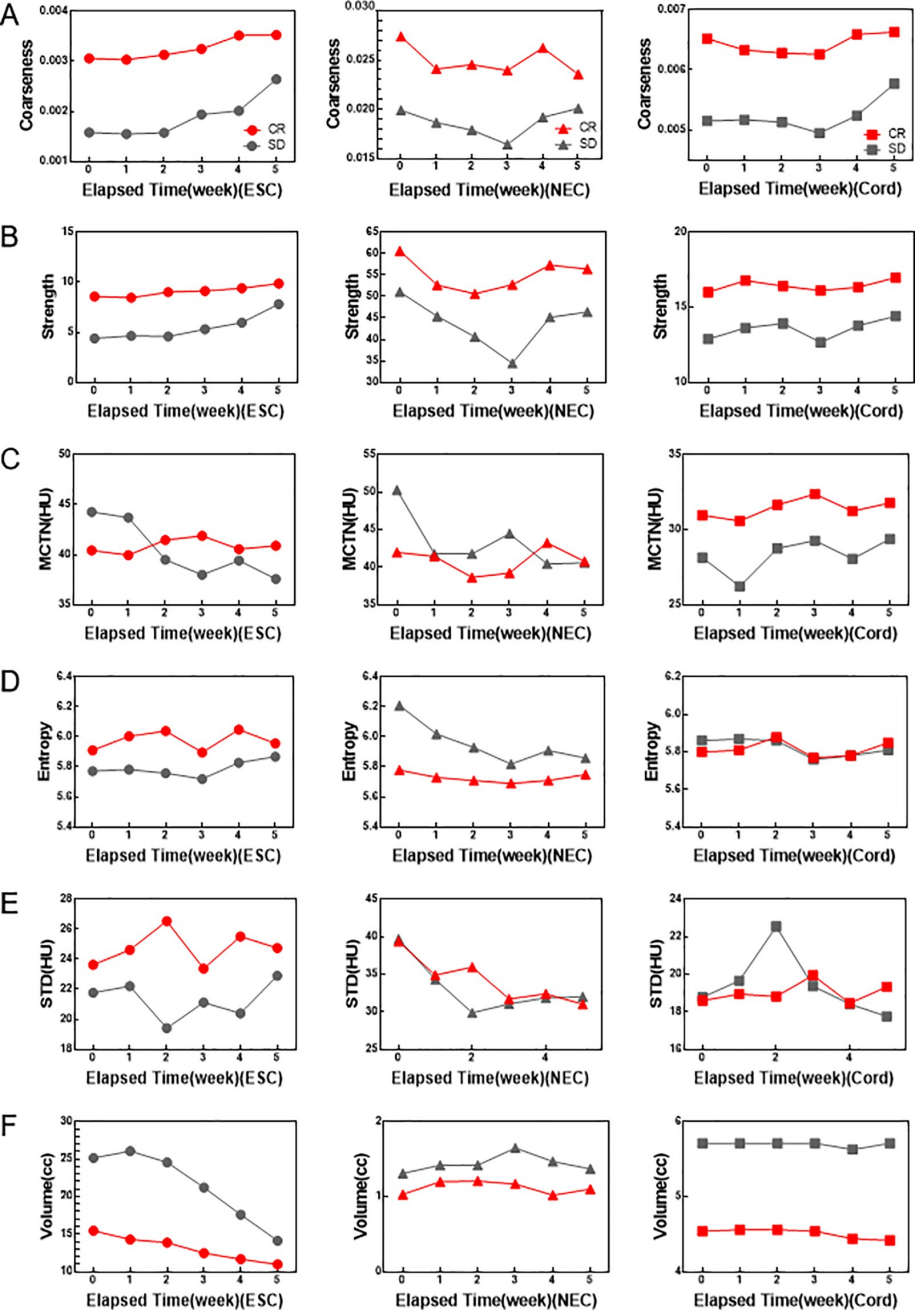
Changes in texture features with treatment. Coarseness (A), strength (B), MCTN (C), entropy (D), STD (E) and volume (F) were observed in patients with good response (CR) and poor response (SD) of ESC, NEC and cord with the elapsed time.

The selected radiomics features in ESC and NEC in patients with good response and poor response changed during treatment; while there were no significant changes in cord. Coarseness and strength in ESC showed an increasing trend; while coarseness and strength in NEC showed a decreasing trend, and volume in ESC was decreased, as shown in Fig 2. The average gross changes of two groups of patients (CR/SD) in coarseness, STD, entropy, and strength between patients with good response and poor response were 0.00047/0.00106(*P* < 0.0001), 1.11/1.13HU (*P* = 0.0007), 0.05/0.10 (*P* = 0.0003), and 1.29/3.42 (*P* < 0.0001) from the beginning to the end of radiation treatment. Among these features, coarseness and strength were related to radiotherapy response using Pearson correlation test (r = 0.8776, P = 0.0216; r = 0.9178, P = 0.0099). A weak, not significant correlation (r = -0.73, *P* = 0.0995) was found between the gross change of MCTN in ESC and treatment response.

### Correlation between texture features and survival time

**Fig 3:**
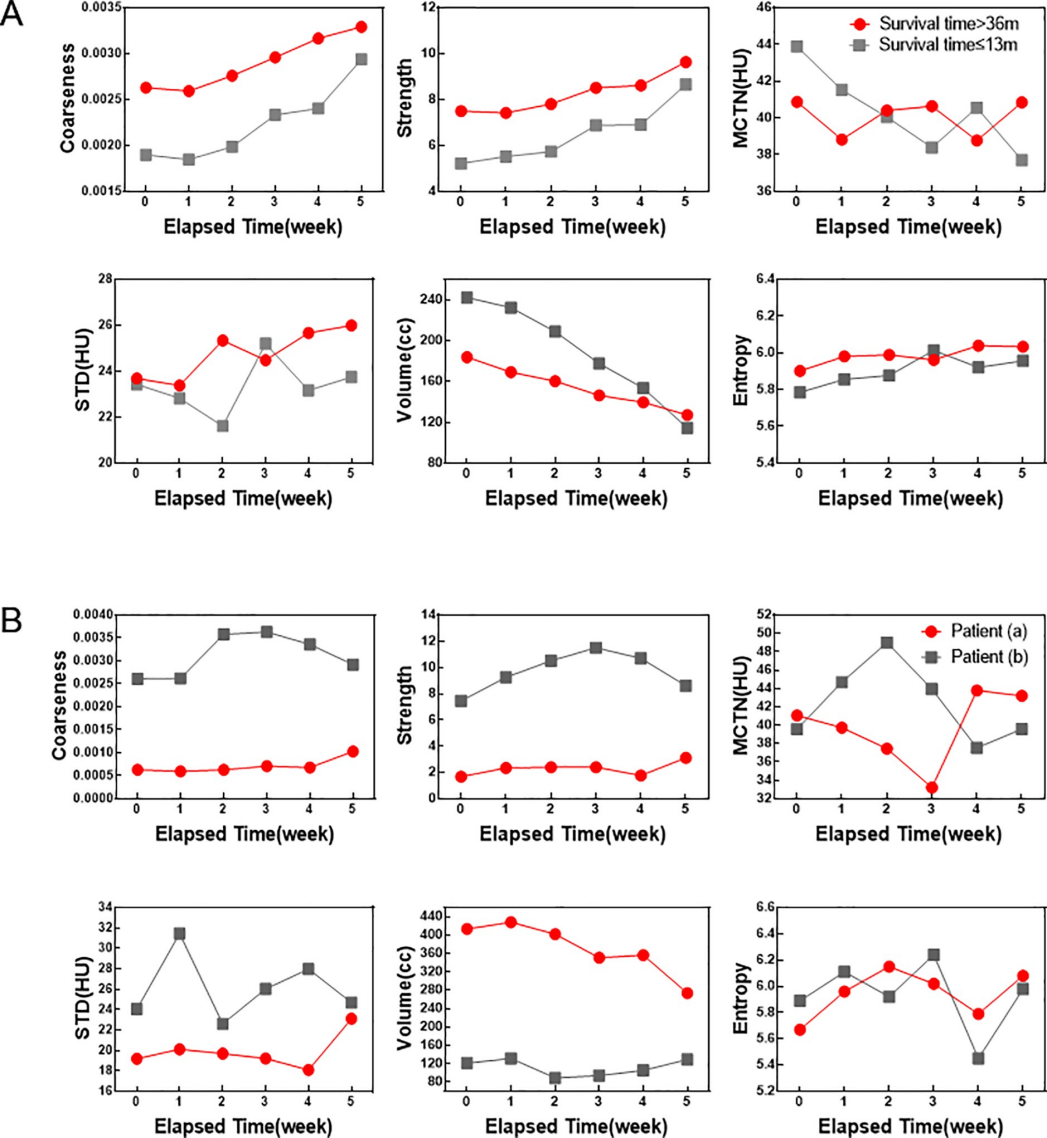
Correlation between texture features and survival. (A)Comparisons for texture features between the two groups with one had a survival greater than 36 months and one less than 13 months along the increase of elapsed time. (B) Analysis of two typical cases, (a) Patient 10 and (b) Patient 41.

The median survival time of patients with good response was 36 months, while patients with poor response was 11.5 months. Patients in this study were divided into two groups for one had a shorter survival time(survival time ≤ 13m) and one had a longer survival time(survival time > 36m). Significant correlation was found between coarseness (r = 0.9572, *P* = 0.0027), strength (r = 0.9917, *P* = 0.0001) and patients survival (Fig 3(A)). Two patients ((a) Patient 10 and (b) Patient 41) were observed particularly. Patient (a) had a poor response but survived for 33 months; while patient (b) responded well to radiotherapy but only survived for 7 months. As shown in the Fig 3(B), the coarseness and strength of patient (a) showed an increasing trend, which was similar to the trend of the two in the patients with a survival time greater than 36 months. While patient (b) did not follow the trend. This suggests that coarseness and strength may predict patients’ survival.

**Fig 4:**
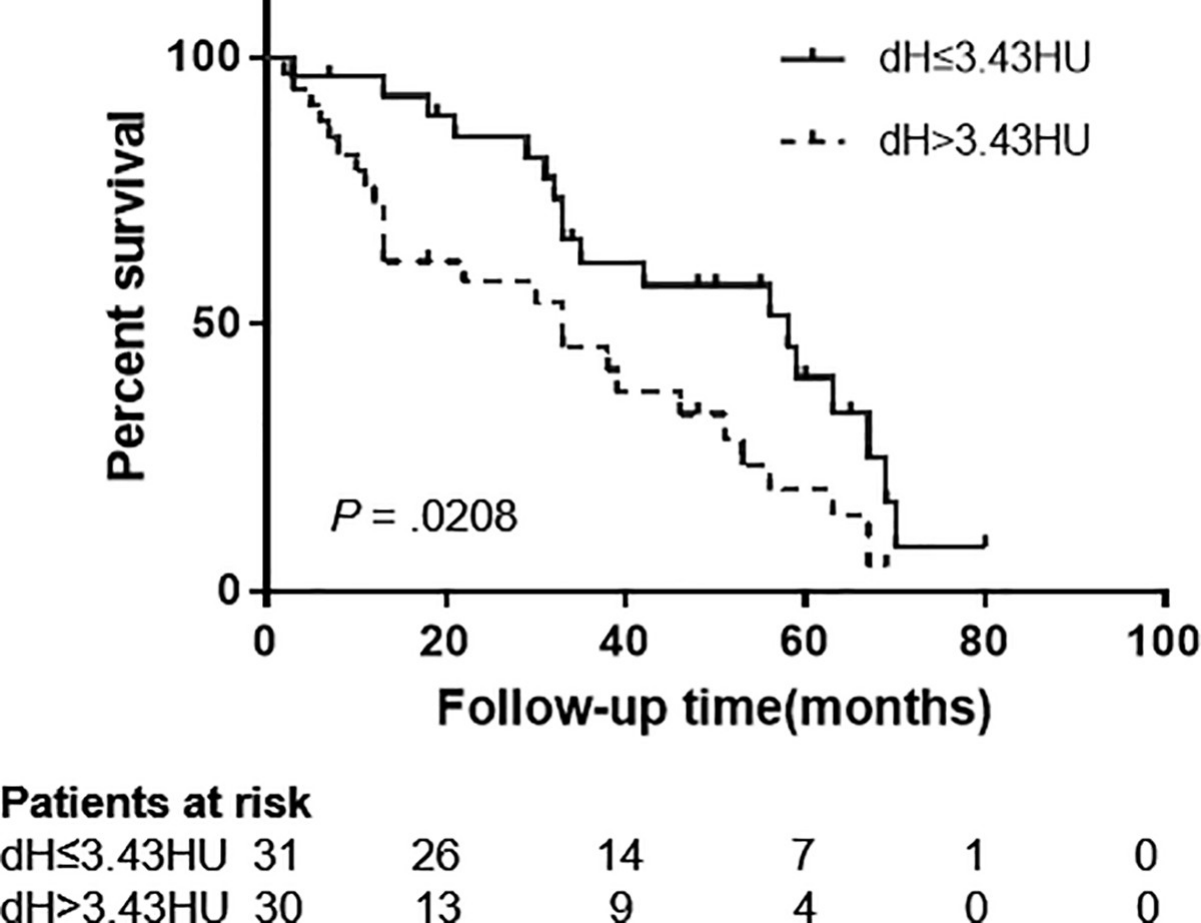
Correlation between MCTN and survival. Kaplan-Meier analysis of the patient percent survival as a function of the median MCTN change for patients studied during the follow-up period.

There was no statistically significant difference in MCTN between patients with long-term and short-term survival time (*P* = 0.7768), as shown in Fig 3(A). Changes of MCTN in ESC (dH) of 61 patients had a median of 3.43HU. As shown in Fig 4, the survival time of patients with lower MCTN changes (less than 3.43HU) was greater than that of larger HU changes (greater than 3.43HU), which was statistically significant by Log-rank tests (*P* = 0.0208). For patients with smaller MCTN changes, the median survival time was 58 months (95% CI 0.9589–3.221), and 33 months (95% CI 0.3104–1.043) for patients with larger MCTN changes. The overall survival rates of patients at 1, 3, and 5 years were 81.97%, 40.98%, and 18.03% respectively.

## Discussion

There was no significant correlation between gross changes of MCTN and treatment response in ESC during RT (r = 0.1429, *P* = 0.8028) in this study, which was different from those reported in lung and pancreatic tumor[11,15]. However, this study showed that the change of MCTN was significantly correlated with survival rate (*P* = 0.0208), which was consistent with the study of Paul et al[12] in lung tumor (*P* = 0.038). In patients with longer survival, the change of MCTN in esophageal carcinoma showed a oscillating pattern (Fig 3(A)). The median change of MCTN was 3.43 HU, and when the change of MCTN was less than that of 3.43HU, the survival rate was higher (Fig 4). Paul et al[12]found a statistically significant increase in survival rates in patients with lung tumor with higher MCTN changes (greater than 30HU).

In addition, coarseness, strength, entropy and STD were strongly correlated with early treatment response during the treatment for ESC (*P* < 0.0001, *P* < 0.0001, *P* = 0.0003 and *P* = 0.0007). These texture features (Fig 3(B)) showed an increasing trend. It was found that there was a strong correlation between survival rate of the patients and the changes of coarseness and strength (r = 0.9572, *P* = 0.0027 and r = 0.9917, *P* = 0.0001). The results are not consistent with the report of Ren et al[7] and may indicate meaningful texture features to predict the radiotherapy response of ESC. They also can be used to predict the radiotherapy response of ESC in the early stage of treatment, and provide an objective measure for treatment of ESC in individual patient basis.

Tumor heterogeneity is a visible character of malignant tumors, which can be quantified by texture analysis. Ganeshan et al[16] found that tumor heterogeneity was an independent prognostic factor for survival by texture analysis of CT images of esophageal carcinoma obtained from PET-CT examinations. The texture parameter, i.e. entropy may be a tumor invasive marker and can predict the survival of patients with esophageal carcinoma. Tixier et al[19] had shown that coarseness can be distinguishable between patients who respond to radiotherapy and those who do not.

Although the change of CT image textures should be attributed to cellular change of tissue during the cancer treatment, the actual mechanism is still not fully clear. Mayer et al[11] suggested that edema and necrosis of tumor tissue occured during radiotherapy, results in changes of MCTN. Feng et al[14] suggest that the increase of tumor blood volume may be the cause of the decrease of MCTN. With the increase of radiation dose, the tumor volume begins to shrink, and the tumor blood volume may begin to increase, which may reduce the tumor density and lead to the decrease of CTN in tumor tissue. In addition, with the increase of radiation dose, some tumor cells die, tumor tissue begins to shrink, eventually CTN is reduced. Cao et al[20] reported that the increase of blood volume in locally controlled tumors was significantly higher than that in patients with local failure. Therefore, the changes of CTN may be related to the changes of tumor blood volume.

Chen et al[15] argued that different structural regions in the tissue respond differently to radiation, and these differences inevitably lead to changes in the shape of the histogram, usually a spread of the peak as indicated by the changes of STD, skewness and kurtosis. In addition, the changes of skewness and kurtosis indicate that CTN histogram gradually changes from non-Gaussian distribution (tissue inhomogeneity) to Gaussian distribution. But the histogram is hard to reflect the spatial structures. Coarseness is likened to granularity in the image and describes the local tumor texture based on differences between each voxel and the adjacent voxels in adjacent image planes[21]. Tixier et al[19] suggested that the changes of tissue texture may be related to the physiological processes related with response of CRT, such as vascularization, perfusion, tumor invasiveness or hypoxia.

Because of the complexity of disease and treatment, there are many other factors that may affect the analysis of data results. Of the 61 patients in this study, 42 received concurrent chemotherapy, and the overall tumor response came from radiotherapy and chemotherapy. These two effects are inseparable as no separate radiation data are available, so they are treated as a whole in this study. Based on our results, there was no significant difference in the weekly changes of coarseness, strength, STD and entropy between patients treated with radiotherapy alone and concurrent chemoradiotherapy (t-test, *P* = 0.7332/0.8747/0.7383/0.1422/0.7066; *P* = 0.6215/0.7180/0.1629/0.9803/0.7179; *P* = 0.4233/0.3313/0.187/0.8082/0.3567 and *P* = 0.8813/0.0581/0.2211/0.3755/0.1148).

There still have spaces to improve in two aspects in this study. One is the sample size, which included only 61 patients. The small sample size prevented us from obtaining a more robust correlation between radiological changes and good response. The second is the accuracy of the definition of tumor tissue. With CT images, tumor region mainly is judged by the extent of invasion by the thickness of esophageal wall, and it was generally considered that > 5 mm was thickening. Therefore, the left, right, anterior and posterior invasion of esophageal lesions can be accurately defined with CT images, but it is quite different from solid tumors in measuring the longitudinal length of esophageal lesions. This difference may be due to inflammation caused by thickening of the esophageal wall and esophageal obstruction at the site of the lesion[22]. In addition, even if the consistency of the daily contours is carefully examined by an experienced radiation oncologist to minimize the contour changes, the subjective judgment of the location of ESC may affect the analysis.

## Conclusions

Coarseness, strength, STD and entropy had significant changes in patients with radiotherapy sensitivity, coarseness and strength were also closely related to patients survival, which may be used to assess treatment response and provide a basis for individualized treatment of esophageal carcinoma. A significant increase in survival rates was shown in patients with lower MCTN changes. In the future, more patient data are needed for more robust model.
